# P043: Re-emergence of influenza a H1N1 in Saudi Arabia

**DOI:** 10.1186/2047-2994-2-S1-P43

**Published:** 2013-06-20

**Authors:** NA Alzahrani, S Al Johani

**Affiliations:** 1 College of Applied Medical Sciences, King Saud bin Abdulaziz University for Health Sciences, Saudi Arabia; 2 Department of Pathology and Laboratory Medicine, King Abdulaziz Medical City, Riyadh, Saudi Arabia

## Introduction

Influenza A H1N1 is a novel influenza virus that is of swine source. Clinical manifestations of this virus vary from mild respiratory symptoms to fatal respiratory or/and cardiovascular complications. The new influenza A H1N1 pandemic was first identified in April 2009 in the United States and Mexico, and then spread globally. In September 10 2010, the World Health Organization announced that the influenza A H1N1 pandemic had moved into the post-pandemic period and is no longer considered a dangerous global disease. On the 15^th^ of August 2010, the Ministry of Health in Saudi Arabia declared that the cases of influenza A H1N1 in 2010 have drastically declined to 874 cases with no deaths or serious complications.

## Objectives

In this study, we will shed light on the number of cases of influenza A H1N1 infections in Saudi Arabia from the months of September to November 2012, and describe the cases where influenza A H1N1 was the cause of death in these patients, and the underlying co-morbidities that may have led to severe complications or death.

## Methods

This is a retrospective study in which we reviewed our laboratory records for cases that were tested for influenza A H1N1 in the months of September, October, and November 2012. All samples were nasopharyngeal aspirates and were tested using The Xpert Flu® assay and performed on Cepheid® GeneXpert medical device system.

## Results

67 patient samples were tested for influenza A H1N1 over the period from September to November 2012. Overall, there were 19 positive samples, 13 of those samples were positive for influenza A H1N1, 6 were positive for influenza A, and none tested positive for influenza B. Two patients were suffering from other co-morbidities and expired as a result of influenza A H1N1 infection.

## Conclusion

Vaccination for influenza A H1N1 is widely available in Saudi Arabia. However, some people are resisting vaccination. This resistance may have caused the re-emergence of influenza A H1N1 in Saudi Arabia. Public awareness should be improved to aware people of the benefits of vaccination and the consequences of not getting vaccinated.

## Disclosure of interest

None declared

